# Integrated Transcriptomic and Metabolomic Analyses Reveal Key Antioxidant Mechanisms in *Dendrobium huoshanense* Under Combined Salt and Heat Stress

**DOI:** 10.3390/plants14152303

**Published:** 2025-07-25

**Authors:** Xingen Zhang, Guohui Li, Jun Dai, Peipei Wei, Binbin Du, Fang Li, Yulu Wang, Yujuan Wang

**Affiliations:** Engineering Technology Research Center of Plant Cell Engineering, Lu’an 237012, China; xingenzhang2023@163.com (X.Z.); guohuili196@163.com (G.L.); wxxydaijun@163.com (J.D.); weipeipei202401@163.com (P.W.); dubb921@126.com (B.D.); lifang2233@163.com (F.L.); wangyulu2025@163.com (Y.W.)

**Keywords:** *Dendrobium huoshanense*, combined salt and high-temperature stress, transcriptome, metabolome, antioxidant systems

## Abstract

Combined abiotic stresses often impose greater challenges to plant survival than individual stresses. In this study, we focused on elucidating the physiological and molecular mechanisms underlying the response of *Dendrobium huoshanense* to combined salt and heat stress by integrating physiological, transcriptomic, and metabolomic analyses. Our results demonstrated that high temperature plays a dominant role in the combined stress response. Physiological assays showed increased oxidative damage under combined stress, accompanied by significant activation of antioxidant enzyme systems (SOD, POD, CAT). Metabolomic analysis revealed significant enrichment of glutathione metabolism and flavonoid biosynthesis pathways, with key antioxidants such as glutathione and naringenin chalcone accumulating under combined stress. Transcriptomic data supported these findings, showing differential regulation of stress-related genes, including those involved in reactive oxygen species scavenging and secondary metabolism. These results highlight a coordinated defense strategy in *D. huoshanense*, involving both enzymatic and non-enzymatic antioxidant systems to maintain redox homeostasis under combined stress. This study provides novel insights into the molecular mechanisms underlying combined stress tolerance and lays the foundation for improving stress resilience in medicinal orchids.

## 1. Introduction

Plants constantly face a variety of abiotic stresses, including heat, drought, and salinity, which can disrupt cellular homeostasis and reduce crop productivity [1,2]. These stresses hinder plant growth and, in severe cases, can even lead to plant death, because they disrupt several critical physiological processes, including water balance, photosynthesis, metabolic pathways, and cell membrane stability [3]. Additionally, the presence of multiple stresses, or compound stresses, is more challenging than the presence of a single stress [4,5,6]. For instance, plants growing in saline soils frequently encounter a dual stressor comprising salinity and drought, and this combined stressor frequently exacerbates water loss, metabolic disorders, and cellular damage in plants, thereby reducing their resilience and leading to a significant decrease in yield [7,8]. Furthermore, the combination of extreme temperatures with other abiotic stresses, such as drought and salinity, exerts a significant additional physiological burden on plants, limiting their growth and productivity [9,10,11].

Drought, salt, and high-temperature stress are common environmental challenges faced by plants during agricultural production. The stresses suffered by crops are often multifactorial, and Elmyhun et al. (2024) found that under the combination of drought and high-temperature stress, the leaves of maize showed significant wilting and increased loss of water, which resulted in plant growth restriction [12,13]. Concurrently, the combined stress of drought and salinity also markedly impeded root development and nutrient uptake in plants, thereby exacerbating growth stagnation [7,14,15]. It is evident that combinations of disparate stress types frequently result in a twofold or even tripled impact on plants. The combination of heat and salt stress has been reported to intensify oxidative damage and alter metabolic responses in many plants. However, the exact nature of this interaction varies among species and remains insufficiently characterized [5,10,16]. In tropical and subtropical regions, the coexistence of extreme high temperatures and saline soils often results in dual stress, and the combined effects of high temperature and salt stress can have a significant impact on plant water uptake, nutrient utilization, and cellular redox balance [15,17,18]. High temperature directly damages cell membranes, enzyme activities, and metabolic processes in plants, whereas salt stress hinders water uptake by reducing the water potential gradient and causing osmotic imbalance in the root zone, ultimately leading to cellular dehydration [19,20,21]. For example, it has been demonstrated that tomato exhibits a considerable inhibition of photosynthesis, leaf desiccation, and wilting under combined salt and high-temperature stress [9,22,23].

In response to salt and high-temperature stress, plants deploy a series of regulatory mechanisms at different levels to facilitate acclimation. However, several studies have investigated the effects of combined salt and heat stress on plants, yet the findings remain inconsistent, highlighting the complexity and species-specific nature of plant responses under such conditions [9,24,25,26,27]. For instance, Li et al. (2011) found that the combination of salt and heat stress specifically induced the expression of proteins such as nucleoside diphosphate kinase 1 and chlorophyll a/b binding protein [25]. Suzuki et al. (2016) demonstrated that the combination of salt and heat stress had a deleterious effect on *Arabidopsis* plants, suggesting a synergistic negative impact of dual stress [27]. In contrast, Rivero et al. (2014) observed that the combination of salt and heat stress provided significant protection for tomato plants from the negative effects of salt stress [26]. This protective effect was attributed to distinct metabolic adjustments, including the accumulation of glycine betaine and phytate. Adding to this complexity, Li et al. (2024) demonstrated that heat plays a dominant role in the combined stress response of tomato, with oxidative phosphorylation acting as a central pathway regulating energy metabolism [9]. Furthermore, studies have shown that exogenous γ-aminobutyric acid as well as symbiotic interactions with arbuscular mycorrhizal fungi and biochar can enhance plant resilience under combined stress [23,28]. Moreover, plants respond to the simultaneous presence of heat and salinity by accumulating flavonoids and activating the mitogen-activated protein kinases (MAPKs) signaling pathways when exposed to combined stress [18,29]. Collectively, these contrasting findings underline the uncertainty in the current understanding of plant responses to combined stresses. Notably, plants may exhibit cross tolerance, in which the response to one stressor enhances resistance to another. This phenomenon underscores the need to investigate the coordinated defense strategies in *D. huoshanense* under simultaneous salt and heat stress.

*Dendrobium huoshanense* is a perennial herbaceous plant of the genus Dendrobium in the family of *Orchidaceae*. It is regarded as the most important of the nine Chinese orchids and is also a National Geographical Indication product of China. However, due to its demanding requirements for the growing environment and climatic conditions, the wild *D. huoshanense* often encounters significant challenges from the combined effects of salt and high temperatures. To better understand the adaptive strategies of *D. huoshanense* under complex environmental conditions, this study integrates physiological, transcriptomic, and metabolomic analyses to systematically investigate its response to simultaneous salt and heat stress. We hypothesize that the co-occurrence of salt and heat stress triggers a distinct and intensified antioxidant response compared to single stress conditions and that metabolic pathways such as glutathione and flavonoid biosynthesis play central roles in mitigating oxidative damage. This work aims to uncover key molecular pathways and potential regulatory mechanisms involved in the cross-tolerance of *D. huoshanense*, ultimately providing a foundation for improving stress tolerance in this valuable medicinal species.

## 2. Results

### 2.1. Biochemical and Physiological Changes in D. huoshanense Under Single and Combined Stress

**Figure 1:**
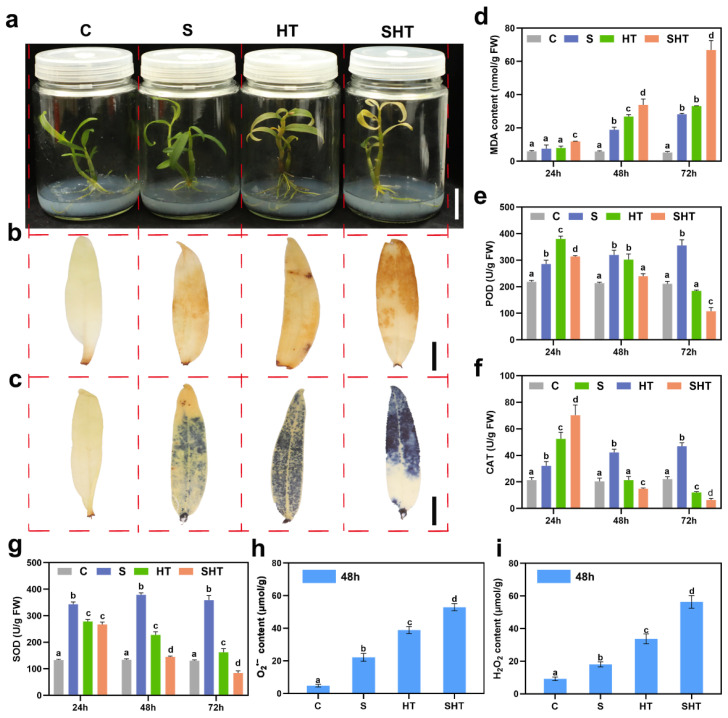
Physiological and biochemical responses of *Dendrobium huoshanense* under salt (S) stress, high-temperature (HT) stress, and combined (SHT) stress. (**a**) Phenotypic appearance of *D. huoshanense* plants after 72 h of exposure to S, HT, or SHT. Scale bar = 2 cm. (**b**) DAB (3,3′-diaminobenzidine) staining of leaves after 48 h of treatment, showing hydrogen peroxide (H_2_O_2_) accumulation as a brown precipitate. Scale bar = 0.5 cm. (**c**) NBT (nitroblue tetrazolium) staining of leaves after 48 h, showing superoxide anion (O_2_^−^) accumulation as a blue precipitate. Red dashed lines indicate separation between different treatment groups for clarity. Scale bar = 0.5 cm. (**d**) Total malondialdehyde (MDA) content in plants under each stress condition. (**e**–**g**) Activities of antioxidant enzymes under each stress: (**e**) superoxide dismutase (SOD), (**f**) catalase (CAT), and (**g**) peroxidase (POD). (**h**,**i**) ROSlevels in leaves after 48 h of treatment: (**h**) superoxide anion content and (**i**) hydrogen peroxide content. Data are presented as mean ± SE (*n* = 3). Different lowercase letters (a, b, c, d) indicate significant differences among treatments based on one-way ANOVA followed by Duncan’s multiple range test (*p* < 0.05).

To evaluate the physiological response of *D. huoshanense* to salt (S), high temperature (HT), and combined (SHT) stress, seedlings were treated accordingly, and samples were collected at 24 h, 48 h, and 72 h. Under salt stress, MDA content and the activities of SOD, POD, and CAT increased gradually over time, suggesting progressive oxidative stress and activation of antioxidant defenses (Figure 1d–g). Under high temperature, MDA levels continued to rise, while antioxidant enzyme activities peaked at 24 h and then declined, indicating impaired defense under prolonged heat. Combined stress caused stronger responses than either stress alone, with higher MDA levels and more drastic changes in enzyme activities. At 48 h, DAB and NBT staining showed stronger brown and blue coloration under stress, especially in the SHT group, indicating higher accumulation of H_2_O_2_ and O_2_^−^ (Figure 1b,c). Quantitative assays confirmed elevated ROS levels under all stress treatments, with the highest levels under SHT (Figure 1h,i). These results show that combined stress leads to more severe oxidative damage than single stresses. Therefore, the 48 h timepoint was selected based on preliminary physiological data showing peak levels of ROS accumulation and antioxidant enzyme activities under stress, thus representing a critical window for capturing transcriptomic and metabolic shifts.

### 2.2. Transcriptome Result

**Figure 2:**
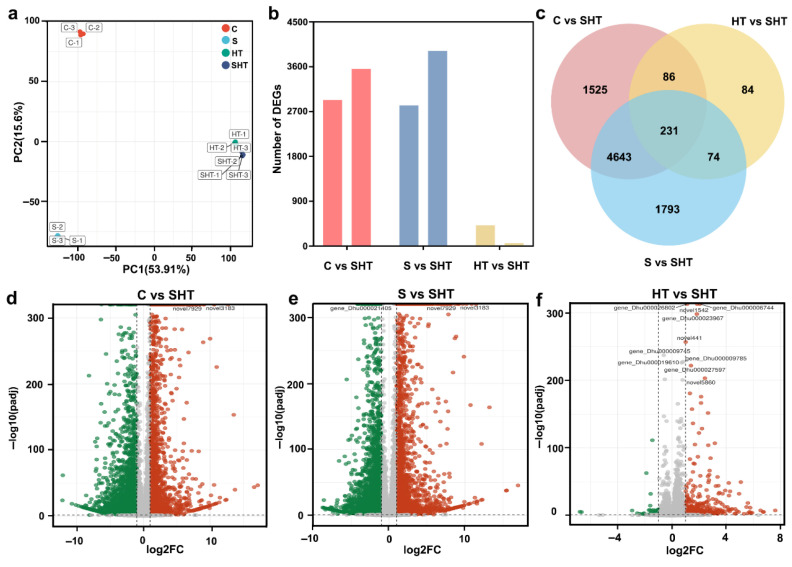
Overview of differentially expressed genes in *D. huoshanense* under different stress conditions. (**a**) Principal component analysis of the transcriptome under control and stress treatments. (**b**) Numbers of genes that are upregulated (left) or downregulated (right) under each stress condition. (**c**) Venn diagram showing the overlap of DEGs among the stress conditions. (**d**–**f**) Volcano plots of gene expression changes for comparisons of control vs. combined stress, salt vs. combined stress, and high-temperature vs. combined stress, respectively. Colors: in volcano plots, red dots indicate significantly upregulated genes, green dots indicate significantly downregulated genes, and gray dots indicate non-significant genes.

Physiological analysis showed that the combination of salt and high-temperature stress had a greater effect on *D. huoshanense* than either stress alone. The transcriptome of *D. huoshanense* under the three stresses was sequenced and analyzed to elucidate the combined effects of salt and high temperature stress at the molecular level. PCA results showed that the four groups could be separated in the PC1 × PC2 score plots, and there was a noticeable grouping trend between SHT and HT (Figure 2a). The number of DEGs was analyzed via transcriptome sequencing. A standard (*p* < 0.05 and Log2FoldChange ≥ 1) was used to screen DEGs. Compared with SHT, 6485 (2929 upregulated 3556 downregulated), 6714 (2822 upregulated and 3919 downregulated), and 475 (415 upregulated 60 downregulated) DEGs were identified in samples of C, S, and HT, respectively (Figure 2b,d–f). A statistical analysis of the DEGs in the three groups (C vs. SHT, S vs. SHT, HT vs. SHT) revealed 231 DEGs that were common to the three groups (Figure 2c).

**Figure 3:**
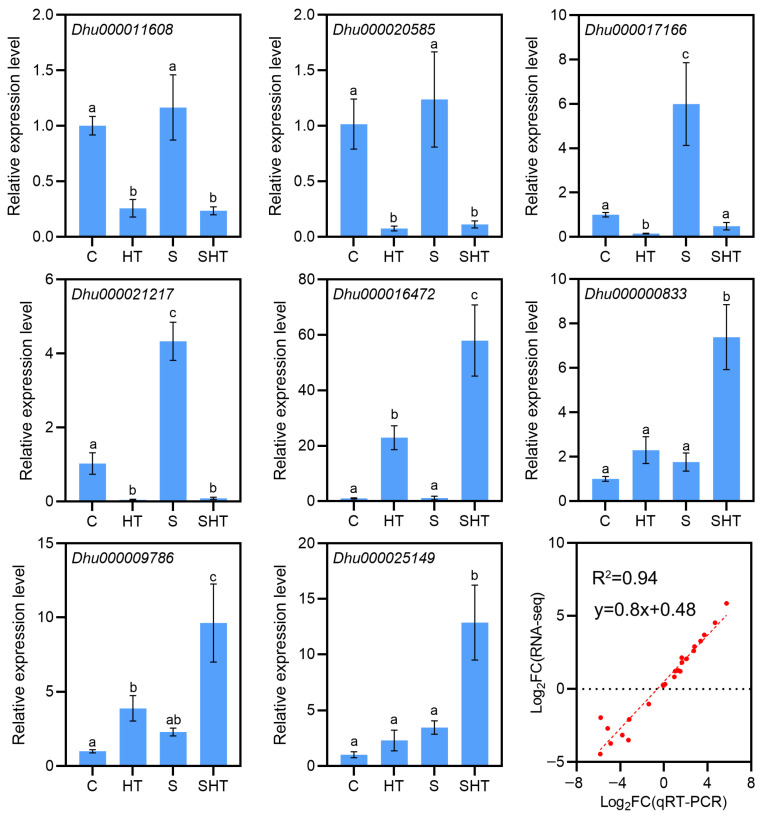
qRT-PCR validation of selected DEGs under the stress treatments. For representative genes, bar charts show the relative expression levels under control, salt, high-temperature, and combined stress conditions. *Actin* is an internal reference gene. The close agreement between the qRT-PCR results and RNA-seq data confirms the reliability of the transcriptomic analysis. The red solid line represents the regression fit between qRT-PCR and RNA-seq data, indicating the close agreement between the two datasets. Different letters (a, b, c, d) indicate statistically significant differences among treatments as determined by one-way ANOVA followed by Duncan’s multiple range test (*p* < 0.05).

To verify the reliability of the RNA-seq data, we selected nine DEGs for qRT-PCR analysis (Figure 3). The qRT-PCR results showed high correlation with the RNA-seq results (R^2^ = 0.94), which confirmed the expression results were reliable (Figure 3).

### 2.3. Weighted Correlation Network Analysis (WGCNA) Analysis

**Figure 4:**
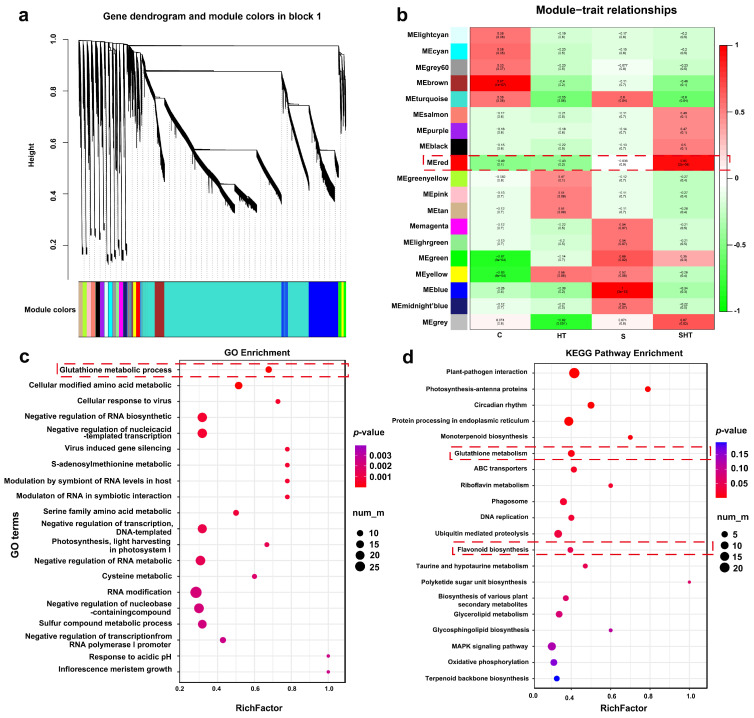
Identification of WGCNA gene co-expression modules and their correlation with stress treatments. (**a**) Clustering dendrogram of DEGs based on topological overlap, with modules labeled in different colors. (**b**) Heatmap showing correlations between module eigengenes and the stress conditions (green and red denote positive and negative correlations, respectively). (**c**) Gene Ontology (GO) enrichment analysis for genes in the red module. (**d**) KEGG pathway enrichment analysis for genes in the red module. Identification and correlation analysis of WGCNA modules. Red dashed lines in (**b**–**d**) highlight key modules or enriched pathways related to glutathione and flavonoid metabolism under stress conditions.

In order to elucidate the molecular response mechanism of *D. huoshanense* to salt, high-temperature, and combined stress, this study employed WGCNA to conduct a comprehensive transcriptome data analysis of the different treatment groups. Initially, the gene expression matrix was subjected to preprocessing, with the objective of retaining the highly variable genes for subsequent network construction. The weighted network was constructed using the soft-thresholding power method (soft-thresholding power), and the co-expression modules were identified through the dynamic tree cutting method (Figure 4a). The correlation analysis was conducted using the module eigengene (ME) with different treatment groups (compound stress, salt stress, high-temperature stress, and control) to determine which modules were significantly related. A correlation analysis was conducted to identify modules with the highest correlation coefficients. The results demonstrated that the red module exhibited the strongest correlation (r = 0.95, *p* < 0.01) with the combined stress treatments, indicating that this module of genes plays a pivotal role in the response of *D. huoshanensis* to combined stress (Figure 4b).

Further functional enrichment analysis was performed on the red module. The GO enrichment results revealed that genes within this module were significantly enriched in several biological processes related to transcriptional repression, including negative regulation of RNA biosynthetic process (GO:1902679), negative regulation of transcription from nucleic acid templates (GO:1903507), negative regulation of transcription from DNA templates (GO:0045892), negative regulation of RNA metabolic process (GO:0051253), and negative regulation of transcription from RNA polymerase II promoter (GO:0000122) (Figure 4c). These findings suggest that combined stress may induce *D. huoshanense* to repress the expression of non-essential functional genes, thereby reprogramming transcription to favor stress response pathways and activate key metabolic pathways involved in stress responses. Notably, processes such as amino acid metabolism (GO:0006520) and glutathione metabolism (GO:0006749) were significantly enriched in this module, exemplifying this regulatory strategy (Figure 4c). This hypothesis is further supported by KEGG pathway enrichment analysis of the red module, which showed significant enrichment in glutathione metabolism and flavonoid biosynthesis pathways (Figure 4d). These metabolites play vital roles in plant stress responses by enhancing the antioxidant system, efficiently scavenging reactive oxygen species, alleviating oxidative stress, and maintaining cellular redox homeostasis and physiological stability.

### 2.4. Analysis of Metabolome Sequencing Results and Functional Annotation of Differential Accumulated Metabolites (DAMs)

**Figure 5:**
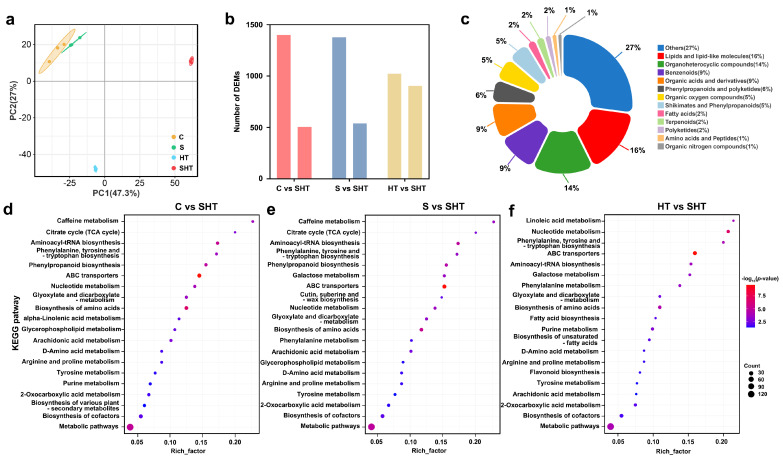
Distribution of differentially accumulated metabolites (DAMs) in *D. huoshanense* under salt, high-temperature, and combined stress. (**a**) Principal component analysis (PCA) of the metabolome under control and stress conditions. (**b**) Numbers of metabolites that show increased accumulation (upregulated, left) or decreased accumulation (downregulated, right) under each stress condition. Red represents control vs. combined stress, blue represents salt vs. combined stress and yellow represents high temperature vs. combined stress (**c**) Classification and proportional representation of DAMs by metabolic category. (**d**–**f**) KEGG pathway enrichment analyses for metabolites in the comparisons of control vs. combined stress, salt vs. combined stress, and high-temperature vs. combined stress, respectively.

Principal component analysis (PCA) of metabolomic profiles revealed clear treatment-specific separation among *D. huoshanense* samples (Figure 5a). The combined salt-and-heat stress group (SHT) was metabolically distinct from both control (C) and single-stress groups (S and HT), indicating that compound stress induces a greater shift in metabolic composition than either stress alone. This observation suggests synergistic effects in metabolite regulation under SHT treatment. Untargeted profiling identified thousands of ion features, from which 1904, 1916, and 1927 DAMs were detected in C vs. SHT, S vs. SHT, and HT vs. SHT comparisons, respectively (Figure 5b). The DAMs covered a wide range of compound classes, including amino acids and derivatives, organic acids, flavonoids, phenylpropanoids, lipids, and polyamines (Figure 5c). KEGG pathway enrichment showed significant overrepresentation of several pathways in SHT-treated plants, including glutathione metabolism, amino acid biosynthesis, ABC transporters, and phenylpropanoid biosynthesis (Figure 5d–f). Glutathione metabolism was enriched in all SHT comparisons (C vs. SHT, S vs. SHT, and HT vs. SHT), implying consistent activation of redox-related defense. In contrast, flavonoid and phenylpropanoid biosynthesis were more prominently enriched in HT vs. SHT (Figure 5f), suggesting that salt stress plays a crucial role in triggering secondary metabolite accumulation under heat conditions. These trends pointed to possible pathway prioritization in response to compound stress, but the full scope and specificity of metabolite responses required further resolution.

**Figure 6:**
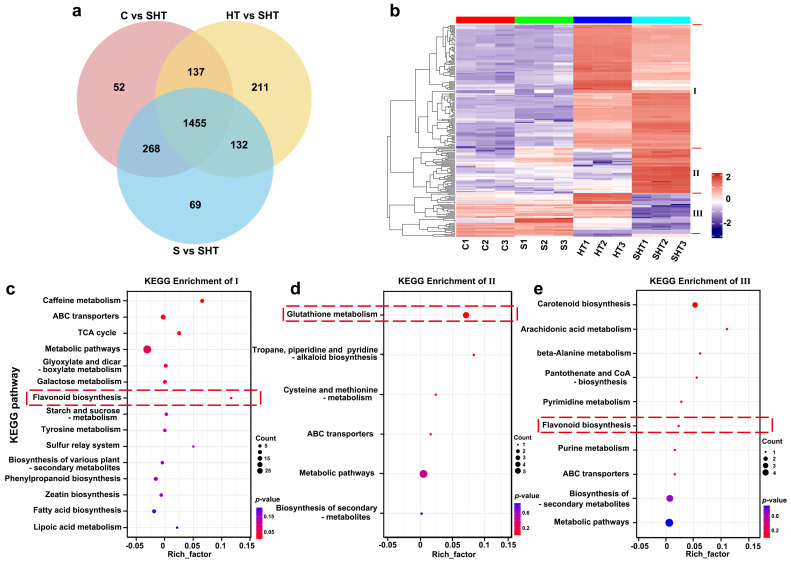
Analysis of DAMs that are common to all three stress comparisons (C vs. SHT, S vs. SHT, and HT vs. SHT). (**a**) Venn diagram showing the overlap of DAMs among the three comparisons (the center subset represents metabolites shared by all three). (**b**) Cluster analysis of the metabolites shared among all three comparisons, revealing three distinct clusters (Class I, II, and III) with different accumulation patterns. (**c**–**e**) KEGG enrichment analyses for the metabolites in Class I, Class II, and Class III, respectively. Red dashed lines in (**c**–**e**) highlight key modules or enriched pathways related to glutathione and flavonoid metabolism under stress conditions.

To dissect the metabolic changes uniquely associated with combined stress, we analyzed DAMs shared across the three SHT-related comparisons (Figure 6a). A total of 1455 overlapping DAMs were identified, representing a core set of metabolites altered specifically under SHT relative to all other treatments. Hierarchical clustering of these shared DAMs revealed three major expression patterns (Figure 6b). Class I metabolites, characterized by strong up-accumulation in SHT, were significantly enriched in glutathione metabolism and amino acid-related pathways (Figure 6c), underscoring their central role in antioxidant defense and osmoprotection under compound stress. Elevated levels of glutathione and related thiol compounds highlight the importance of redox buffering in SHT conditions. Class II metabolites, which were downregulated under SHT, were associated with pathways such as carotenoid and energy metabolism (Figure 6d), potentially reflecting the tradeoff between stress response and growth-related functions. In contrast, Class III metabolites showed more complex patterns and were significantly enriched in flavonoid and phenylpropanoid biosynthesis (Figure 6e). Taken together, these results demonstrate that glutathione metabolism and flavonoid biosynthesis pathways are among the most responsive to combined salt and heat stress in *D. huoshanense*.

### 2.5. Conjoint Analysis of Transcriptomics and Metabolomics

To further elucidate the molecular mechanisms underlying the response of *D. huoshanense* to combined salt and high-temperature stress, we conducted an integrative analysis of DEGs and DAMs across the C vs. SHT, S vs. SHT, and HT vs. SHT comparison groups. The results indicated that the SHT treatment induced distinctive changes in several biological processes compared with the other three groups (Table S2). Pathway enrichment analysis of the overlapping DEGs and DAMs across these comparisons revealed that most of the shared pathways were associated with metabolic processes, including the biosynthesis of various secondary metabolites (stilbenoid, diarylheptanoid, and gingerol biosynthesis; general secondary metabolite biosynthesis; caffeine metabolism; flavonoid biosynthesis; phenylpropanoid biosynthesis), lipid metabolism (cutin, suberine, and wax biosynthesis; alpha-linolenic acid metabolism), amino acid-related metabolism (glutathione metabolism), and membrane transport (ABC transporters). Among these pathways, glutathione metabolism and flavonoid biosynthesis are particularly noteworthy due to their critical roles in regulating antioxidant capacity, which is essential for coping with salt, heat, and combined stresses.

**Figure 7:**
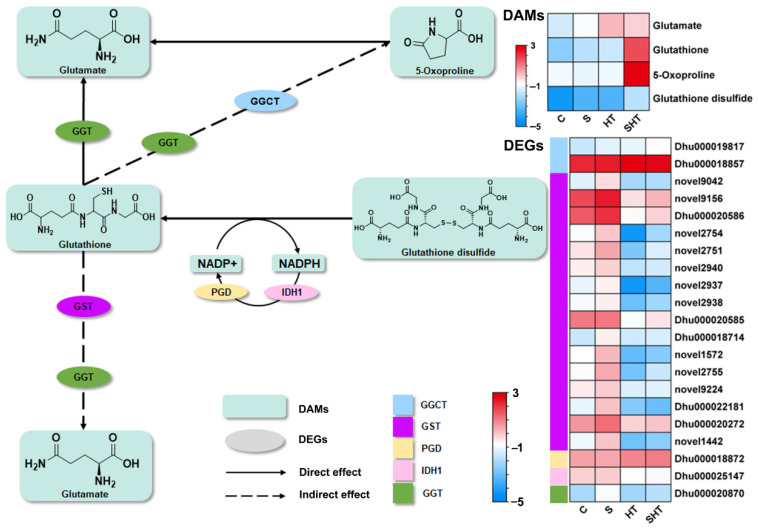
Differential changes in genes and metabolites involved in the glutathione (GSH) metabolism pathway under stress. Enzymes in the pathway are labeled by their gene symbols: GGCT (gamma-glutamylcyclotransferase), GST (glutathione S-transferase), PGD (6-phosphogluconate dehydrogenase), IDH1 (isocitrate dehydrogenase), and GGT (gamma-glutamyltranspeptidase). Changes in gene expression (DEGs) and metabolite levels (DAMs) are indicated as log_2_ fold changes relative to the control.

Glutathione metabolism represents a major detoxification and redox-regulatory pathway. In this study, four key metabolites in this pathway (glutamate, glutathione, 5-oxoproline, and glutathione disulfide) were significantly more abundant in all stress treatments relative to the control, with the SHT group exhibiting the highest accumulation. This result suggests that glutathione plays an essential role in alleviating oxidative stress and enhancing stress tolerance under combined salt and heat exposure. Interestingly, the expression levels of genes encoding glutathione S-transferase (GST) were downregulated in the SHT group compared to the C and S groups (Figure 7), despite the simultaneous increase in glutathione and related metabolites. This may suggest a shift in antioxidant strategy, in which *D. huoshanense* prioritizes glutathione biosynthesis and enzymatic ROS scavenging (e.g., by POD and CAT) over GST-mediated detoxification. Moreover, GST activity may also be regulated at the post-transcriptional or post-translational level, which could explain the discrepancy between gene expression and metabolite accumulation.

**Figure 8:**
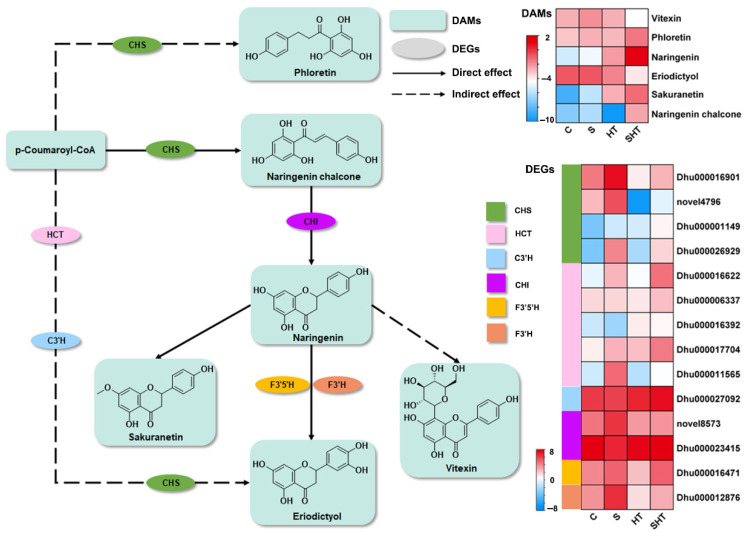
Differential changes in genes and metabolites involved in the flavonoid biosynthesis pathway under stress. Enzymes are labeled by their gene symbols: CHS (chalcone synthase), HCT (shikimate O-hydroxycinnamoyl transferase), C3′H (flavonoid 3′-hydroxylase), CHI (chalcone isomerase), F3′5′H (flavonoid 3′,5′-hydroxylase), and F3′H (flavonoid 3′-hydroxylase). The changes in gene expression and in flavonoid metabolite accumulation are shown as log_2_ fold changes compared to the control.

In parallel, flavonoid biosynthesis also showed strong transcriptional and metabolic responses under stress. The levels of key antioxidant metabolites, naringenin and naringenin chalcone, were significantly elevated in the SHT group compared to the other three groups (Figure 8). These flavonoids are known for their ability to scavenge ROS and protect cells from oxidative injury. Naringenin chalcone, a critical intermediate in the flavonoid biosynthesis pathway, not only contributes to redox regulation but also plays vital roles in defense against pathogens and herbivores [30,31]. Gene expression patterns supported these findings. The expression of chalcone synthase (CHS), a key enzyme initiating flavonoid biosynthesis, was lowest in the HT group but highest in the S group, suggesting that salt stress promotes while heat stress represses flavonoid biosynthesis. In the SHT group, CHS expression showed an overall upregulation trend, indicating potential synergistic activation of flavonoid synthesis under combined stress conditions. Moreover, chalcone isomerase (CHI), which catalyzes a downstream step in flavonoid biosynthesis, exhibited consistently high expression across all stress treatments [32], underscoring its indispensable role in maintaining flavonoid production under adverse conditions.

## 3. Discussion

### 3.1. Integrated Stress Response of D. huoshanense to Combined Salt and Heat Stress

Due to interactions between stress sources, combined stresses can be viewed as a novel type of stress, and when facing multiple stress combinations, plants may show unique phenotypic and physiological changes because, in general, compared to single stresses, plants exhibit more severe phenotypic changes under combined stresses [16,33,34,35,36,37,38,39,40,41,42,43,44,45,46]. In this study, *D. huoshanense* showed more pronounced phenotypic and physiological changes under combined salt and high-temperature stress than under either stress alone, suggesting a synergistic effect. Similar observations have been made in *Arabidopsis*, where low-level individual stresses caused negligible effects, but their combination led to significant damage, suggesting a synergistic effect rather than a merely additive one. This implies that the presence of one stressor may amplify the detrimental impact of the other, resulting in an outcome greater than the sum of individual effects [37]. Physiological indicators such as CAT and POD activity in the SHT group were more similar to those under heat stress alone, and transcriptome and metabolome PCA also showed clustering of SHT with HT (Figure 1, Figure 2 and Figure 5). These results suggest that high temperature may dominate the plant’s response in the combined stress condition. This pattern aligns with previous findings in tomato plants [9], where high temperature had a more immediate impact on above-ground tissues than salt, which must first be absorbed by roots and transported to shoots. Thus, the rapid and direct effects of heat stress may position it as the leading factor in this stress combination [38,39,40].

### 3.2. Antioxidant and Secondary Metabolism Are Coordinately Activated Under Combined Stress

The antioxidant mechanism is one of the core strategies plants use to adapt to environmental stress [3,41,42]. This study combined comprehensive analyses of physiology, transcriptomics, and metabolomics to clearly demonstrate the crucial role of antioxidant pathways in *D. hooshanense*’s response to combined salt and high-temperature stress. Combined stress induces the accumulation of ROS in plants, which leads to lipid peroxidation and damage to proteins and DNA. Therefore, the main function of the antioxidant mechanism is to eliminate excess ROS in the body and protect plant cells from oxidative damage.

Oxidative stress is a common outcome of environmental stressors, and plants have evolved complex antioxidant systems to mitigate ROS-induced damage. In *D. huoshanense*, the activities of SOD, POD, and CAT were markedly elevated under SHT stress, indicating activation of the enzymatic antioxidant system. These enzymes detoxify ROS like superoxide radicals and hydrogen peroxide, preventing damage to lipids, proteins, and DNA [43,44,45]. In support of these findings, physiological measurements provided direct evidence of oxidative stress and antioxidant responses under combined treatment. The levels of hydrogen peroxide (H_2_O_2_) and superoxide anion (O_2_^−^) were significantly elevated in SHT-treated plants, indicating excessive ROS accumulation. Correspondingly, histochemical staining with DAB and NBT revealed pronounced brown and blue coloration in leaves under SHT, consistent with enhanced ROS production. These visual and quantitative indicators reinforce that oxidative stress is a major challenge under combined stress, and the observed activation of antioxidant enzymes represents an essential protective mechanism.

Beyond enzyme-based defenses, plants deploy non-enzymatic antioxidants, among which glutathione plays a central role. Glutathione (GSH) directly scavenges ROS and participates in redox signaling [44,46,47,48]. In this study, four metabolites including glutamate, glutathione, 5-oxoproline, and glutathione disulfide accumulated significantly under SHT stress. This suggests that glutathione metabolism is robustly activated to maintain cellular redox homeostasis. Interestingly, while several glutathione-related metabolites such as glutathione, glutathione disulfide, and 5-oxoproline were significantly upregulated under combined stress, most GST-encoding genes showed reduced or unchanged expression levels. This apparent mismatch may reflect a stress-induced shift in antioxidant strategy, where *D. huoshanense* prioritizes the synthesis of glutathione and the activity of ROS-scavenging enzymes (e.g., POD, CAT) over GST-mediated detoxification. Additionally, GST proteins are known to undergo complex post-transcriptional and post-translational regulation, so mRNA abundance may not fully represent actual enzymatic activity. This suggests that GST activity may be regulated at the post-transcriptional or post-translational level, such as through phosphorylation or redox-based modifications.

In addition to glutathione, flavonoids represent a key component of the plant’s secondary antioxidant network. These compounds not only scavenge ROS but also stabilize membranes, regulate osmotic potential, and interact with hormonal pathways [49,50,51,52,53]. For example, *Ginkgo biloba* seedlings can tolerate low levels of soil salinity stress by regulating the biosynthesis of non-flavonoid compounds, thereby better responding to environmental stresses [54]. Over-expression of *MsFLS13* in *Medicago sativa* enhances flavonol accumulation, antioxidant capacity, osmotic balance, and photosynthetic efficiency, improving the plant’s tolerance to saline–alkaline stress [55]. Under combined stress, we observed substantial increases in naringenin chalcone and quercetin, both of which possess strong antioxidant activities and are precursors in the flavonoid biosynthesis pathway. Naringenin chalcone is a core intermediate with functions in both defense and development [30,31], while quercetin has been shown to alleviate both salt- and heat-induced cellular damage [56,57]. Though the expression of CHS and CHI, two key enzymes in the flavonoid biosynthesis pathway, varied among treatments, CHI maintained high expression across all stress conditions, suggesting its stable contribution to flavonoid synthesis under stress. The regulatory divergence in CHS expression, especially the SHT-induced recovery from heat-inhibited levels, further supports the idea that *D. huoshanense* adjusts flavonoid metabolism through multifaceted control mechanisms under complex stress. These findings highlight the potential of flavonoid biosynthetic genes as candidate targets for future breeding or genetic engineering efforts to enhance stress tolerance in orchids.

### 3.3. WGCNA Analysis Highlights Key Antioxidant Modules

To identify key regulatory modules involved in the stress response, WGCNA was performed using transcriptomic data. The red module showed a strong positive correlation with antioxidant-related metabolic pathways. GO and KEGG enrichment analysis revealed that this module was significantly enriched in glutathione metabolism and flavonoid biosynthesis, aligning well with the physiological and metabolomic data. These findings suggest that this module may serve as a hub for coordinating antioxidant responses at the transcriptional level. Notably, the enrichment of multiple GST- and flavonoid-related genes in this module further emphasizes the central roles of these pathways in mediating the plant’s response to combined stress.

## 4. Materials and Methods

### 4.1. Plant Material and Treatment

Three-month-old *D. huoshanense* tissue culture seedlings were cultivated in a controlled environment under a 12 h light/12 h dark cycle at 25/22 °C (day/night), 70% relative humidity, and a photosynthetic photon flux density of 150 μmol·m^−2^ × s^−1^. Healthy and uniform seedlings were selected for stress treatments. Salt stress (S) was simulated by transferring seedlings to MS medium supplemented with 200 mM NaCl, and this NaCl concentration was selected based on previous studies. Treatments ranging from 150 to 250 mM NaCl in *D. officinale* and *D. catenatum* have been shown to trigger physiological responses such as ROS accumulation and antioxidant enzyme activation without causing immediate lethality [58]. High-temperature stress (HT) was imposed by placing seedlings in a 42 °C incubator. For combined stress (SHT), seedlings were simultaneously exposed to 200 mM NaCl and 42 °C. Sampling was conducted at 24, 48, and 72 h, and all treatments were conducted in three biological replicates.

### 4.2. Biochemical and Physiological Analysis

Leaves of *D.huoshanense* were sampled at 24, 48, and 72 h after treatment for biochemical and physiological analyses. Malondialdehyde (MDA) content and superoxide dismutase (SOD) activity were measured using commercial kits (Jiangsu Bioisco Biotechnology Co., Ltd., Nanjing, China), following the manufacturer’s protocols. Peroxidase (POD) and catalase (CAT) activities were determined using assay kits (Nanjing Jiancheng Bioengineering Institute, Nanjing, China). To evaluate oxidative stress accumulation, 3,3′-diaminobenzidine (DAB) and nitroblue tetrazolium (NBT) staining were performed on leaves collected at 48 h post-treatment. DAB staining was performed with a horseradish peroxidase DAB chromogenic kit (Beyotime Biotechnology, Shanghai, China), and NBT staining was carried out using an alkaline phosphatase NBT chromogenic kit (Beyotime Biotechnology, Shanghai, China), following the manufacturers’ protocols. The contents of hydrogen peroxide (H_2_O_2_) and superoxide anion (O_2_^−^) were also quantified at 48 h post-treatment. H_2_O_2_ levels were measured using a hydrogen peroxide assay kit (Nanjing Jiancheng Bioengineering Institute, Nanjing, China), while superoxide anion content was assessed using a superoxide anion inhibition and generation detection kit (Nanjing Jiancheng Bioengineering Institute, Nanjing, China), following the protocols provided by the manufacturer. All physiological measurements were performed with at least three biological replicates for each treatment.

### 4.3. Transcriptome Sequencing and WGCNA Analysis

Total RNA was extracted from each sample using the TRIzol Reagent Mini Kit (Qiagen, Hilden, Germany) following the manufacturer’s protocol. RNA quality was assessed using a NanoDrop spectrophotometer (Thermo Fisher Scientific Inc., Waltham, MA, USA) and an Agilent 2100 Bioanalyzer (Agilent Technologies, Palo Alto, CA, USA). Samples with an RNA integrity number (RIN) > 9 were used for library construction. Poly(A) mRNA was isolated using the NEBNext^®^ Poly(A) mRNA Magnetic Isolation Module (New England Biolabs Inc., Ipswich, MA, USA), and libraries were prepared using the NEBNext^®^ Ultra™ RNA Library Prep Kit for Illumina^®^ (New England Biolabs Inc., Ipswich, MA, USA) according to the manufacturer’s instructions. Multiplexed libraries were sequenced on the Illumina HiSeq platform (Illumina, San Diego, CA, USA).

Raw reads were first evaluated for quality using FastQC v0.11.9, and then adapters and low-quality reads were removed using Trimmomatic (v0.39). Clean reads were aligned to the *D. huoshanense* reference genome (version: *D.huoshanense*.v20200604.chromosome) using STAR (v2.7.9a) with default parameters. Gene-level read counts were obtained using featureCounts (v2.0.1), and downstream differential gene expression analysis was performed using DESeq2 (v1.30.1). Genes were considered differentially expressed if they met the following criteria: |log_2_(fold change)| ≥ 1 and FDR < 0.05, based on three biological replicates. The use of FDR (false discovery rate) accounts for multiple testing corrections given the large number of genes analyzed. The principal component analysis (PCA) was performed using the prcomp function in R (v4.2.1). Gene Ontology (GO) and Kyoto Encyclopedia of Genes and Genomes (KEGG) enrichment analyses for DEGs were conducted using clusterProfiler (v3.18.1).

Weighted Gene Co-expression Network Analysis (WGCNA) was carried out using the WGCNA R package (v1.69) as described by Langfelder and Horvath (2008) [59]. The input was the normalized count matrix filtered for low-expression genes. A soft thresholding power was chosen to construct a scale-free network, and modules of co-expressed genes were identified by dynamic tree cutting method. Correlation between modules and physiological traits was calculated to identify trait-related modules. GO and KEGG enrichment analyses were then performed for each key module using clusterProfiler. Network visualization was conducted using Cytoscape (v3.9.1).

### 4.4. Metabolite Extraction, Quantification, and Metabolomics Analysis

For metabolite extraction, 100 μL of each sample was mixed with 400 μL of extraction solution (methanol/acetonitrile, 1:1, *v*/*v*), containing deuterated internal standards. The mixture was vortexed for 30 s, sonicated in a 4 °C water bath for 10 min, and then incubated at −40 °C for 1 h to precipitate proteins. Following incubation, samples were centrifuged at 12,000 rpm (RCF = 13,800× *g*, rotor radius = 8.6 cm) for 15 min at 4 °C. The supernatant was collected and transferred into fresh glass vials for subsequent analysis. A quality control (QC) sample was prepared by pooling equal volumes of all supernatants. Liquid chromatography–mass spectrometry (LC-MS/MS) analysis was performed using a Vanquish UHPLC system (Thermo Fisher Scientific) coupled with an Orbitrap Exploris 120 mass spectrometer. Separation was conducted on a Waters ACQUITY UPLC BEH Amide column (2.1 mm × 50 mm, 1.7 μm). The mobile phase consisted of (A) 25 mmol·L^−1^ ammonium acetate and 25 mmol·L^−1^ ammonia hydroxide in water (pH = 9.75), and (B) acetonitrile. The autosampler temperature was maintained at 4 °C, and the injection volume was 2 μL.

The mass spectrometer operated in both positive and negative electrospray ionization (ESI) modes with the following source parameters: sheath gas flow rate 50 Arb, auxiliary gas flow rate 15 Arb, capillary temperature 320 °C, spray voltage +3.8 kV (positive mode) or −3.4 kV (negative mode), full MS resolution of 60,000, and MS/MS resolution of 15,000. Data-dependent acquisition (DDA) mode was used with stepped collision energy (SNCE) set to 20/30/40. Xcalibur software (version 4.3, Thermo Fisher Scientific) was used for instrument control and data acquisition. Raw LC-MS data were converted into mzXML format using ProteoWizard (version 3.0.11781) and processed with an in-house R-based workflow built on the XCMS package for peak detection, extraction, alignment, and integration. Metabolite annotation was performed using the BiotreeDB (version 3.0). Differentially accumulated metabolites (DAMs) were identified and quantified using the metaX software (version 3.0.11781) package, based on the following criteria: fold change ≥ 2 (|log2FC| ≥ 1), false discovery rate (FDR) < 0.05, and variable importance in projection (VIP) value ≥ 1.

### 4.5. Integrated Transcriptomic and Metabolomic Analysis

To explore the molecular response of *D. huoshanense* to combined salt and high-temperature stress, transcriptomic and metabolomic datasets were integrated using Kyoto Encyclopedia of Genes and Genomes (KEGG) pathway-based analysis. Differentially expressed genes and differentially accumulated metabolites were identified from the comparisons between the control (C) and combined stress (SHT) groups. KEGG enrichment analyses were independently performed for both DEGs and DAMs to identify significantly affected biological pathways. Key KEGG pathways shared between transcriptomic and metabolomic datasets were then identified through cross-comparison, highlighting core metabolic processes responsive to combined stress. Similar integrative analyses were conducted for the S vs. SHT and HT vs. SHT comparison groups to further dissect the stress-specific regulatory patterns. Finally, key DEGs and DAMs were mapped to their corresponding pathways, and interaction networks were constructed using ChiPlot (https://www.chiplot.online, accessed on 20 October 2024, the version used was the most recent online version available at the time of access) to visualize the relationships between transcripts, metabolites, and enriched pathways.

### 4.6. Validation of RNA-Seq Data by qRT-PCR

To validate the RNA-seq results, eight differentially expressed genes (DEGs) were selected for quantitative real-time PCR (qRT-PCR) analysis. First-strand cDNA was synthesized using the Evo M-MLV RT Premix for qRT-PCR (Accurate Biotechnology Co., Ltd., Changsha, China) following the manufacturer’s instructions. Gene-specific primers were designed using Primer3Plus (http://www.primer3plus.com/, accessed on 10 May 2025). qRT-PCR reactions were performed using the FastStart Essential DNA Green Master Mix (Roche, Basel, Switzerland) according to protocols described previously [60]. The Dendrobium *Actin* gene was used as an internal reference for normalization. Each reaction was conducted with three technical replicates per cDNA sample. Relative gene expression levels were calculated using the 2^−ΔΔCT^method [61]. Primer sequences are listed in Supplementary Table S1.

### 4.7. Data Analysis

All physiological and biochemical data were analyzed using one-way analysis of variance (ANOVA) with IBM SPSS Statistics 16.0 (SPSS Inc., Chicago, IL, USA) to assess differences among treatment groups (control, salt, heat, and salt + heat). When significant differences were found (*p* < 0.05), Duncan’s multiple range test was applied as a post hoc analysis to determine pairwise differences between groups. Data are presented as mean ± standard error (SE) from three biological replicates. Normality of the data was assessed using the Shapiro–Wilk test, and all datasets met the assumption of normality. Significant differences are denoted by different letters in the figures.

## 5. Conclusions

**Figure 9:**
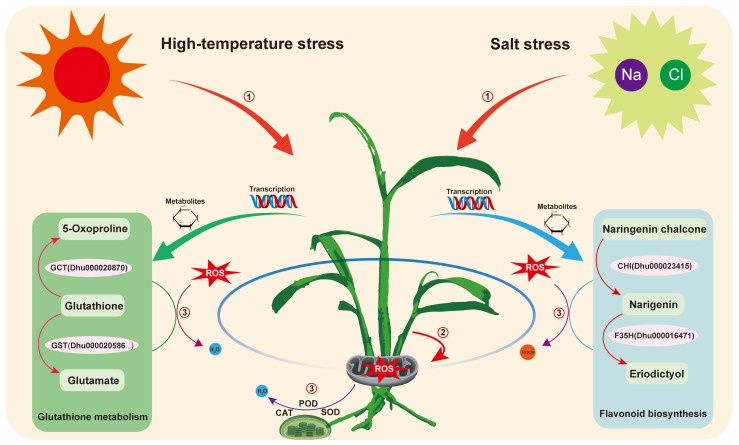
Proposed regulatory mechanism of *D. huoshanense* in response to combined salt and high-temperature stress. Key stress-response pathways are highlighted in different colors in the schematic diagram. Combined stress leads to excessive accumulation of reactive oxygen species such as singlet oxygen (^1^O_2_), superoxide (O_2_^−^), and hydroxyl radicals (OH). The elevated ROS levels activate antioxidant defense systems, in which enzymes such as SOD, CAT, and POD play a key role in ROS detoxification, accompanied by metabolic adaptations including enhanced glutathione metabolism and flavonoid biosynthesis.

This study reveals the coordinated response mechanisms of *D. huoshanense* under combined salt and high-temperature stress through integrated physiological, transcriptomic, and metabolomic analyses. High temperature emerged as the dominant factor shaping the stress response, inducing pronounced changes in gene expression and metabolite accumulation. The plant activates both enzymatic antioxidants (such as SOD, POD, and CAT) and non-enzymatic antioxidants (including glutathione and flavonoids) to alleviate oxidative damage. Metabolomic profiling highlighted significant enrichment in glutathione metabolism and flavonoid biosynthesis, while transcriptomic data demonstrated dynamic regulation of key stress-responsive genes such as *GST*, *CHS*, and *CHI*. As shown in Figure 9, we propose a regulatory model summarizing these findings, in which ROS accumulation under combined stress activates antioxidant enzyme activity and triggers downstream metabolic reprogramming. This model illustrates how D. huoshanense employs a multifaceted antioxidant strategy—through the activation of enzymatic defenses (e.g., SOD, POD, CAT) and the accumulation of antioxidant metabolites (e.g., glutathione and flavonoids)—to mitigate ROS accumulation and enhance tolerance to abiotic stress. Together, these findings provide molecular targets such as CHI, CHS, and glutathione metabolic enzymes, which could be explored in future genetic engineering or molecular breeding efforts to enhance stress resilience in *D. huoshanense*. The significantly altered metabolite profiles may also serve as potential biomarkers for stress tolerance screening.

## Data Availability

The raw sequence data reported in this paper have been deposited in the Genome Sequence Archive (Genomics, Proteomics & Bioinformatics 2021) in National Genomics Data Center (Nucleic Acids Res 2022), China National Center for Bioinformation/Beijing Institute of Genomics, Chinese Academy of Sciences (GSA: CRA021090) that are publicly accessible at https://bigd.big.ac.cn/gsa/browse/CRA021090 (accessed on 9 December 2024).

## Supplementary Materials

The following supporting information can be downloaded at: https://www.mdpi.com/article/10.3390/plants14152303/s1, Table S1: Primers used in this study; Table S2: KEGG pathways from integrated transcriptomic and metabolomic analyses in C vs. SHT, S vs. SHT, and HT vs. SHT.

## Abbreviations

The following abbreviations are used in this manuscript:

ROS	Reactive oxygen species
CAT	Catalase
SOD	Superoxide dismutase
POD	Peroxidase
MDA	Malondialdehyde
